# Utility of Ultrasonography for Diagnosing an Ectopic Testis With Torsion: A Case Report

**DOI:** 10.7759/cureus.66008

**Published:** 2024-08-02

**Authors:** Takayuki Fujii, Hiroyuki Satoh, Atsuko Sato, Yoshiaki Ishizuka, Mizuki Izawa

**Affiliations:** 1 Department of Urology, Tokyo Metropolitan Children's Medical Center, Fuchu-shi, JPN; 2 Department of Pediatric Surgery, Kagawa University, Takamatsu, JPN

**Keywords:** ectopic testis, cryptorchidism, undescended testis, testicular torsion, testicular ectopia

## Abstract

Since ectopic twisted testes are a rare condition, correctly and opportunely diagnosing them preoperatively is difficult and can result in testicular necrosis. We report a clinical case of a twisted ectopic testis that was diagnosed preoperatively by ultrasonography, and the testis could be rescued. A generally healthy 13-year-old boy was referred to our Urology Department after experiencing a painless swelling in the left inguinal region two weeks before, and mild exercise-induced pain in the same area one week before the referral. The mild pain persisted without worsening. On examination, a mildly tender swelling was present in the left inguinal region. The left half of the scrotum was empty; however, the right testis was normal in size and position. Ultrasonography revealed that the left spermatic cord was present within the inguinal canal and was directed superficially, with spiral twisting. The left testis was located above the inguinal canal, with normal echogenicity, but was smaller than the right normal testis (right testis, 41 × 28 × 16 mm; left testis, 18 × 18 × 8 mm). Power Doppler ultrasound showed normal blood flow in the left testis. Consequently, we diagnosed an ectopic testis with torsion. Intraoperative examinations confirmed the presence of the testis in the left superficial inguinal pouch. Although the testis had twisted five and a half turns (1980°) clockwise at the level of the superficial inguinal pouch, ischemia was not evident. Orchidopexy of both testes was performed, and the left testicular size was maintained after surgery. If swelling is present in the inguinal region and no testis is found in the scrotum, an ectopic testis should be considered in the differential diagnosis. Preoperatively diagnosing an ectopic, twisted testis by ultrasonography alone is difficult. However, we used ultrasonography effectively to diagnose the ectopic testis preoperatively by tracking the spermatic cord and confirming the torsion of the testis.

## Introduction

An ectopic testis is located away from the normal route of testicular descent and outside the ipsilateral hemiscrotum [1]. An ectopic twisted testis is extremely rare. Since the incidence of ectopic twisted testis is low, correctly diagnosing it preoperatively is difficult, thus causing delayed diagnosis and potential testicular necrosis [2]. We report a case of an ectopic twisted testis that we diagnosed preoperatively by ultrasonography, and the testis could be preserved.

## Case presentation

A generally healthy 13-year-old boy was referred to our Urology Department after experiencing a painless swelling in the left inguinal region two weeks prior, and mild exercise-induced pain in the same area one week before the referral. The mild pain persisted without worsening.

**Figure 1:**
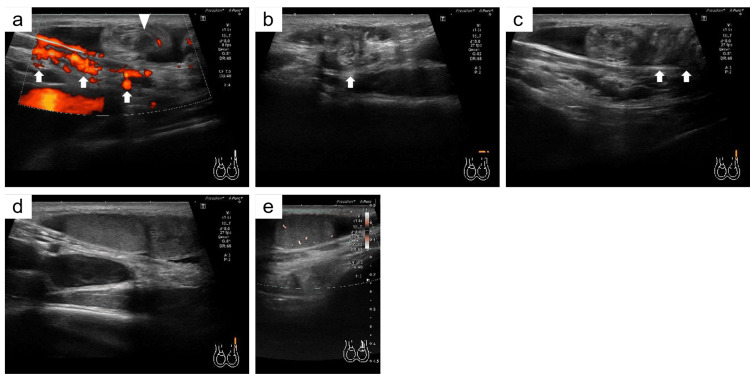
Ultrasonography images a) Arrows show the spermatic cord within the inguinal canal. The triangle shows the spermatic cord above the inguinal canal. b) The spermatic cord is directed toward the body surface (arrow). c) Arrows show multiple twists in the spermatic cord. d) The left testis shows normal echogenicity. e) Power Doppler ultrasound shows preservation of blood flow in the left testis.

On examination, a swelling (2 × 2 cm) with mild tenderness was present in the left inguinal region. The left half of the scrotum was empty; however, the right testis was normal in size and position. Ultrasonography revealed that the left spermatic cord was present within the inguinal canal and was directed superficially, with spiral twisting (Figures 1a-1c). The left testis was located above the inguinal canal, with normal echogenicity, but was smaller than the right normal testis (right testis, 41 × 28 × 16 mm; left testis, 18 × 18 × 8 mm; Figure 1d). Power Doppler ultrasound showed normal intratesticular blood flow (Figure 1e).

Consequently, we diagnosed an ectopic testis with torsion. Previous medical checkups had not revealed any abnormalities in the position of the testes, and the patient could not remember whether the testes had ever been located in the scrotum. Thus, it was unclear when this patient had developed an ectopic testis. We explained to the patient's guardians that, because the patient may have suffered for a long time, spermatogenic disorders or malignancies could be present. We proposed left orchiectomy as a treatment option; however, the patient's family preferred to preserve the affected testis. Thus, we decided to perform a testicular biopsy in an attempt to preserve the testis.

**Figure 2:**
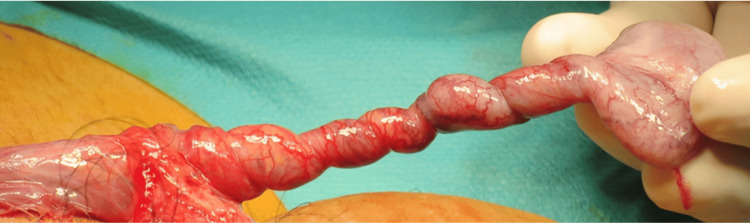
Intraoperative image Image showing the testis twisted five and a half turns (1980°).

Intraoperative examinations confirmed the presence of the testis in the left superficial inguinal pouch. Although the testis had twisted five and a half turns clockwise (1980°) at the level of the superficial inguinal pouch, ischemia was not evident (Figure 2).

We performed a left testicular biopsy and orchidopexy for both testes. Histopathology revealed only a few spermatogonia within the seminiferous tubules, indicating maturation arrest. No malignancy was detected, and the left testicular size was maintained for more than one year after surgery.

## Discussion

An ectopic testis is a rare congenital anomaly, accounting for 5% of undescended testes [3]. The superficial inguinal pouch is the most common location for ectopic testes, accounting for half of such cases [1]. After leaving the external inguinal ring, the testis is inverted and lies between the external oblique muscle and Scarpa's fascia. Possible causes of ectopic testes include abnormal attachment of the vas deferens, blockage of the descent route to the scrotum, or abnormalities of the genitofemoral nerve [3]. Ectopic testes with torsion are extremely rare but can reportedly occur in the superficial inguinal fossa, perineum, or abdominal wall [4-6]. Trauma or spastic neuromuscular disorders may predispose individuals to torsion of an ectopic testis [7]. In the present case, exercise may have contributed to the torsion, since the patient’s pain occurred during exercise.

Hence, it was necessary to distinguish whether the patient had an undescended testis or an ectopic testis. In the case of undescended testis, even if the testis lies in the inguinal region, swelling is rarely observed and is only detectable on palpation. Additionally, ultrasonography revealed that the affected testis was located above the inguinal canal, a finding that is not observed in undescended testes. Thus, we diagnosed this case as an ectopic testis. Furthermore, torsion of an ectopic testis in the superficial inguinal pouch may be diagnosed as an incarcerated inguinal hernia owing to the presence of inguinal swelling. Moreover, a twisted ectopic testis could present with abdominal symptoms, which may be confused with acute appendicitis or intestinal obstruction [8]. Confirming the presence of testes in the scrotum bilaterally is important for differential diagnosis.

Prompt surgical treatment for ectopic testes is recommended because they will not naturally descend into the scrotum [1]. Consensus is lacking regarding whether prophylactic contralateral orchidopexy should be performed on the normal testis when treating a contralateral ectopic twisted testis [5].

Preoperatively diagnosing torsion of an ectopic testis by ultrasonography alone is difficult [4-6]. However, the “tracking-the-cord technique” can be successfully used to detect a nonpalpable testis, including an ectopic testis, by ultrasonography [9]. Accordingly, the presence of the spermatic cord in the inguinal canal guides us to the ectopic testis. In this case, we used ultrasonography effectively to diagnose the ectopic testis preoperatively by tracking the spermatic cord and confirming testicular torsion. Ultrasonography should be performed to differentiate an inguinal swelling from an inguinal hernia. Additionally, ectopic testes can be diagnosed by carefully monitoring the course of the spermatic cord.

Ectopic testes are exposed to a higher temperature than descended intrascrotal testes and may develop histopathological changes similar to those seen in undescended testes [10]. A histopathologic analysis reported that testes located in the superficial inguinal pouch had a lesser number of germ cells per tubule than normally positioned testes [11]. Moreover, such testes can become malignant or cause infertility [1]. In this case, histopathology confirmed testicular maturation arrest. Histopathological changes suggest that ectopy could have been present for a considerable time. Furthermore, the affected testis was smaller than the contralateral normal testis and was suspected to be underdeveloped. This finding also suggests a long period of elevation.

## Conclusions

If an inguinal swelling is present and no testis has descended into the scrotum, an ectopic testis should be considered as a differential diagnosis. Ultrasonography should be performed to differentiate an inguinal swelling from an inguinal hernia. Additionally, ectopic testes can be diagnosed by carefully monitoring the course of the spermatic cord.
